# Menrath ulcers in cats: four cases (2014‐2023)

**DOI:** 10.1111/jsap.13828

**Published:** 2025-01-12

**Authors:** K. Whybrow, T. Hernon, M. Pilot

**Affiliations:** ^1^ Langford Veterinary Services Langford UK

## Abstract

**Objectives:**

To report the clinical presentation, treatment and outcomes of four cats diagnosed with Menrath ulcers causing significant oral haemorrhage.

**Materials and Methods:**

For all cats, data on signalment, history, physical examination, treatment and outcomes were collected by reviewing medical records. Information regarding outcomes was collected from communication logs between primary care veterinarians and owners, and the original case clinicians after discharge of the patient from the hospital.

**Results:**

Four cats were included. All patients survived to discharge. Follow‐up outcomes were available between 1 and 8.5 months post discharge. Post‐operative complications were classified according to The Accordion Severity Classification of Post‐operative Complications. Two of the four patients had recurrence of oral haemorrhage post‐ligation originating from the major palatine artery. One classified as a severe complication, due to requiring revision surgery of the ipsilateral major palatine artery. The other classified as mild, since the patient was managed conservatively. Additionally, one patient was documented to have developed an acquired palatal defect post‐operatively.

**Clinical Significance:**

This is the largest case series of Menrath ulcers to date and the first to describe post‐operative complications including acquired palatal defect and recurrence of oral haemorrhage from the original ulcer despite ligation of the ipsilateral major palatine artery.

## INTRODUCTION

**FIG 1:**
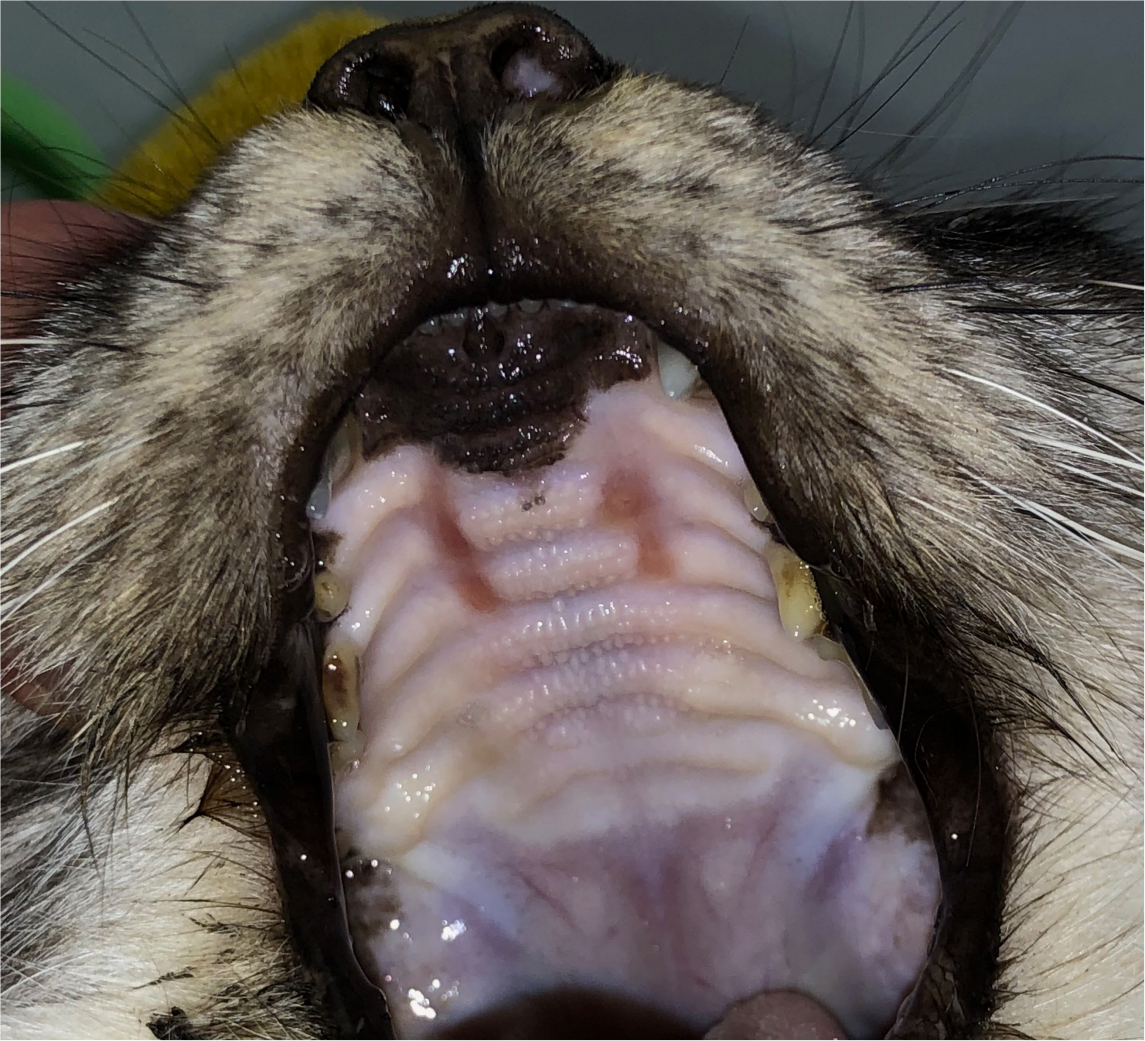
Bilateral Menrath ulcers on the rostral hard palate of a cat.

Menrath ulcers in cats, also described as palatine erosions in the literature, are uncommon (Hall, 2020). Typically, Menrath ulcers have a well‐demarcated appearance and are found unilaterally or bilaterally between the dental arcade and midline of the rostral hard palate (Whitley et al., 2017; Wildgoose, 1990) (Fig 1). Progressive erosion can result in profuse spontaneous haemorrhage.

Menrath ulcers can be life‐threatening due to arterial bleeding from the major palatine arteries and associated branches (Hall, 2020; Menrath & Miller, 1995). These erosive lesions are thought to be caused by repetitive abrasion of the hard palate by the tongue's filiform papillae when cats overgroom. Pruritic skin disease caused by flea infestation is often described as the primary cause of overgrooming in these patients (Bailey et al., 2007; Mantelli et al., 2023). Although limited research is available, intervention is often recommended to prevent continued or recurrence of arterial bleeding. The surgical intervention involves ligating the major palatine artery, unilaterally or bilaterally (Hall, 2020; Menrath & Miller, 1995; Wildgoose, 1990).

The literature regarding this condition is sparse, with few individual case reports and descriptions available (Klinprathum et al., 2022; Mantelli et al., 2023; Whitley et al., 2017; Wildgoose, 1990). This is the largest case series describing the presentation and treatment of Menrath ulcers in cats to date. This case series describes several complications not previously reported with surgical management of Menrath ulcers and describes variations in surgical approach not previously defined in the literature.

## MATERIALS AND METHODS

### Medical records search

In order to include all the cats that were presented to Langford Vets small animal referral hospital between January 1, 2010, and December 31, 2023, with a Menrath ulcer, the following searches were performed. Hospital practice management software (RxWorks) was searched by two operators independently in 2023 using keywords “palatal erosion”, “Menrath” and “Menrath Ulcer”. The search identified four cats. All four records were assessed for eligibility, and no records were excluded.

All cases were presented for further investigation and treatment of moderate‐to‐severe regenerative anaemia associated with an oral lesion on the rostral hard palate. Summaries of the clinical findings and for all cases can be found in Table 1; summaries of surgical approach, reported complications and revision surgery can be found in Table 2.

**Table 1:** Summary of initial clinical findings for four cats with Menrath ulcers

	Case 1	Case 2	Case 3	Case 4
Signalment	9‐year‐old male neutered, domestic shorthair (DSH)	3.5‐year‐old male neutered, DSH	3‐year‐old male neutered, Maine Coon	7‐year‐old male neutered, DSH
Presenting signs	Oral haemorrhage Unproductive retching Weight loss Anorexia	Oral ulceration Anaemia	Oral haemorrhage Melaena	Oral haemorrhage Lethargy Anorexia
Physical examination	Respiratory rate (RR) 44 breaths per minute, heart rate (HR) 186 beats per minute and temperature 38.6°C Pale mucous membranes Grade III/VI parasternal systolic heart murmur Live fleas	Respiratory rate (RR) 36 breaths per minute, heart rate (HR) 200 beats per minute and temperature 38.5°C Pale pink mucous membranes Grade II/VI parasternal systolic heart murmur Live fleas	Respiratory rate (RR) 48 breaths per minute, heart rate (HR) 220 beats per minute and temperature 38.7°C Pale mucous membranes Live fleas	Respiratory rate (RR) 44 breaths per minute, heart rate (HR) 180 beats per minute and temperature 36.7°C Pale mucous membranes Gallop sound Live fleas
Oral examination	Unilateral, small focal mucosal purple lesion left side of midline, rostral hard palate	Unilateral, focal erosive area right side of midline rostral hard palate, approximately 4 mm	Bilateral well‐circumscribed ulcers either side of midline of rostral hard palate	Unilateral, superficial ulceration right side of rostral hard palate
Abnormalities on initial haematology and serum biochemistry	Haematocrit (HCT) 12.6 L/L (RI 25.0 to 45.00) Reticulocytosis 180,000/μL (RI 0/μL) Alanine aminotransferase (ALT) 105 IU/L (RI 15 to 45 IU/L) Creatinine 105 μmol/L (RI 133 to 175 μmol/L)	Packed cell volume (PCV) 9% and total solids (TS) 62 g/L HCT 7.5 L/L (RI 25.0 to 45.00) Reticulocytosis 50,000/μL (RI 0/μL) Total protein 47 g/L (RI 77 to 91 g/L) Phosphate 2.04 mmol/L (RI 0.95 to 1.55 mmol/L)	PCV 10% and TS 64 g/L HCT 15.8 L/L (RI 25.0 to 45.00) Reticulocytosis 260,000/μL (RI 0/μL) Urea 13.6 mmol/L (RI 6.5 to 10.5 mmol/L)	PCV 9% and TS 60 g/L HCT 13.2 L/L (RI 25.0 to 45.00) Reticulocytosis 120,000/μL (RI 0/μL) ALT 186 IU/L (RI 15 to 45 IU/L)

**Table 2:** Summary of surgical intervention, complications and revision surgery for four cats with Menrath ulcers

	Case 1	Case 2	Case 3	Case 4
Surgical technique	Left major palatine artery ligated. Suture material and size not recorded	Right major palatine artery cauterised. Approached by incision into lingual side of right maxillary teeth and reflecting oral mucosa to identify palatine artery. Oral mucosal flap sutured 4‐0 polyglactin 910	Bilateral major palatine artery ligation using encircling 2‐0 polydioxanone sutures. Ulcers cauterised monopolar diathermy	Right major palatine artery ligated using encircling 3‐0 polydioxanone suture. Ulcer cauterised monopolar diathermy
Reported complications/follow up time	Severe complication – Major oral haemorrhage 2 weeks post‐surgery requiring blood transfusion and revision surgery	Possible complication (severity not verified) – palatal defect 2 months post‐surgery incidentally, no concurrent clinical signs documented. 1 month post palatal defect recorded by primary care vet no clinical signs of rhinitis or oral haemorrhage detected. Long‐term follow‐up not available	Mild complication – Minor oral haemorrhage 7 months post‐surgery	None 2 months post‐surgery
Revision surgery	U‐shaped incision made around ulcer caudally. Encircling ligatures biting down to hard palate to enclose vascular branches entering from medial, lateral and caudal aspects. Suture material and size not recorded	None	None	‐
Reported complications/follow‐up time	None reported at 3 months post‐revision surgery	‐	‐	‐

### Data extracted from medical records

For all cats, data on signalment, presenting signs, physical examination, treatment and outcomes were extracted from medical records and recorded on an Excel spreadsheet. Additional follow‐up data was collected from communication logs recorded between the primary case clinicians and the owner or primary care veterinarian after discharge from the hospital using the hospital practice management software. Complications were classified according to Follette et al. (2020) contraction of The Accordion Severity Classification of Post‐operative Complications.

## RESULTS

### Case 1

A 9‐year‐old, male neutered, domestic short‐haired (DSH) cat was presented for investigation of a 2‐week history of oral haemorrhage, unproductive retching, anorexia and weight loss. On physical examination, the patient was mildly tachypnoeic with a respiratory rate of 44 breaths per minute. Other vital parameters were within normal limits. Additionally, the cat had pale mucous membranes, a grade III/VI parasternal systolic heart murmur with an intermittent gallop sound and flea infestation. Oral examination documented a small focal purple mucosal lesion to the left of midline on the rostral hard palate consistent with a Menrath ulcer.

Haematology on presentation documented low haematocrit (HCT) 12.6 L/L [reference interval (RI) 25.0 to 45.0 L/L] and reticulocytosis 180,000/μL (RI 0/μL) consistent with a moderate, regenerative anaemia. Evaluation of a blood smear documented polychromasia, anisocytosis and plentiful platelets with evidence of platelet clumping. Serum biochemistry demonstrated a mild increase in alanine aminotransferase (ALT) activity 105 IU/L (RI 15 to 45 IU/L) thought to be due to poor oxygen supply to the liver and a mild decrease in creatinine concentration 105 μmol/L (RI 133 to 175 μmol/L) consistent with reduced muscle mass, the remaining biochemistry results were unremarkable.

Following assessment, the patient received a packed red blood cell transfusion increasing packed cell volume (PCV) from 14% to 20%. Flea treatment was prescribed, selamectin (Stronghold; Pfizer). Additionally, the patient was treated for congestive heart failure with 1 mg/kg frusemide (Frusedale; Dechra) twice daily. Cardiac changes identified on echocardiography were thought to be consistent with volume overload secondary to chronic anaemia or cardiomyopathy. Repeat echocardiography in 3 months was recommended. The cat was hospitalised for 4 days in which no further oral haemorrhage was documented and PCV remained at 20%. The patient was discharged with the diagnosis of a healing Menrath ulcer secondary to chronic dermatopathy.

Twenty‐four hours post‐discharge, the patient was readmitted due to recurrence of oral haemorrhage. PCV had decreased to 12%, and surgical intervention was recommended. The cat was taken to surgery, and due to the left‐sided location of the oral ulcer, the left major palatine artery was ligated under general anaesthesia. The suture material used was not recorded. A subsequent increase in PCV to 25% over the next 4 days was documented. The patient was discharged with 2.5 mg cetirizine hydrochloride to be administered once daily for 7 days for pruritus.

Two weeks post‐discharge, the patient was represented for continued excessive grooming and recurrence of significant oral haemorrhage. The left‐sided Menrath ulcer was still present, and no new ulceration was noted on repeat oral examination.

Haematology documented a moderate anaemia, HCT 15.3 L/L (RI 25.0 to 45.0) and absence of reticulocytes, suspected pre‐regenerative at this stage. As a result of the anaemia and associated clinical signs of tachycardia (heart rate 230 beats per minute) and tachypnoea (respiratory rate 50 breaths per minute), a haemoglobin (Oxyglobin) transfusion was administered. Revision surgery was recommended and performed. A U‐shaped incision was made around the ulcer caudally. Several encircling sutures were placed biting down to palatal bone to enclose vascular branches entering from lateral, medial or caudal aspects. The suture material used was not recorded. To manage persistent overgrooming, 1 mg/kg prednisolone (Prednidale; Dechra) once daily was initiated for ongoing use.

Three months post‐operatively, the patient was reported by the owner via email update to be doing well, coat quality had improved and weight increased back to a normal range. No evidence of repeat oral haemorrhage had been noted. Recommendations to the primary care vet included weaning prednisolone to lowest effective dose for controlling pruritus and reducing frusemide to 1 mg/kg once daily for 3 weeks as no further signs of congestive heart failure were documented whilst closely monitoring respiratory rate.

### Case 2

A 3.5‐year‐old, male neutered, DSH cat was presented for investigation of a 5‐day history of anaemia and oral ulceration. On presentation, vital parameters were within normal limits. Physical examination identified pale pink mucous membranes, a grade II/VI parasternal systolic heart murmur and flea infestation. On oral examination, a focal erosive area on the right side of the rostral hard palate, approximately 4 mm in length, was identified.

Haematology documented low HCT 7.5 L/L (RI 25.0 to 45.0 L/L) and reticulocytosis 50,000/μL (RI 0/μL). PCV was 9% and total solids (TS) 62 g/L. Blood smear evaluation identified macrocytosis, anisocytosis and polychromasia. These findings were consistent with severe anaemia with some evidence of regeneration. Serum biochemistry documented mild hypoproteinaemia 47 g/L (RI 77 to 91 g/L) consistent with recent haemorrhage and mild hyperphosphatemia 2.04 mmol/L (RI 0.95 to 1.55 mmol/L), which was non‐specific. As the patient was cardiovascularly stable, no blood transfusion was administered.

Surgical intervention was recommended for the suspected Menrath ulcer. An incision was made on the palatal side of the right maxillary teeth from the level of the canine to molar region. The palatal mucosa was reflected medially so that the right palatine artery could be identified and cauterised using monopolar electrocautery. The flap was sutured using horizontal mattress and simple continuous sutures 4‐0 polyglactin 910 (Vicryl; Johnson and Johnson). Twenty‐four hours post‐surgery, PCV had increased from 9% to 13%.

Two days post‐operatively, the patient was discharged with 1 mg/kg Prednisolone every other day for 5 days to manage pruritus and Lotilaner (Credelio; Elanco Animal Health) ongoing to treat and prevent flea infestation.

**FIG 2:**
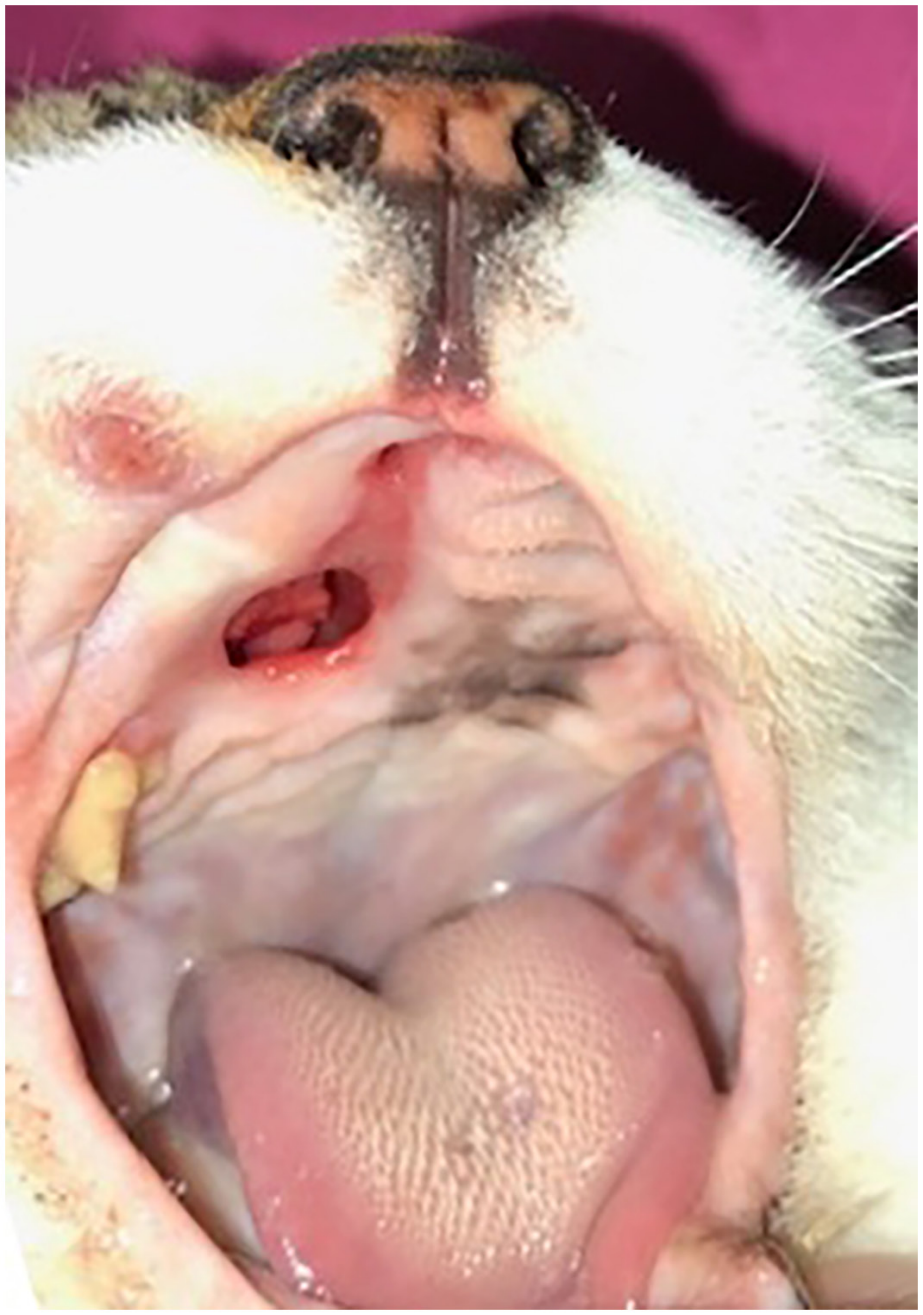
Palatal defect to right side of rostral hard palate in the region of previous Menrath ulcer documented 2 months post‐operatively.

Two months post‐operatively, on clinical examination by the primary care veterinarian, a palatal defect was identified in the same region as the previous Menrath ulcer measuring 5 mm × 8 mm (Fig 2). The patient was reported to have mild clinical signs related to the palatal defect including occasional sneezing when eating. Investigations by the primary care veterinarian showed the anaemia had resolved with haematological parameters back within normal limits and no further oral haemorrhage had been documented. Revision surgery was discussed but declined due to the absence of significant clinical signs and financial constraints. It was decided to proceed with conservative management. Three months post‐operatively, an update from the owner via telephone reported the patient was reported to be doing well with no current clinical signs of rhinitis or oral haemorrhage.

### Case 3

A 3‐year‐old, male neutered, Maine Coon cat was presented with a 2‐day history of oral haemorrhage and melena. On presentation, the patient was tachycardic (heart rate 220 beats per minute), tachypnoeic (respiratory rate 48 breaths per minute) and normothermic. Physical examination identified pale mucous membranes and flea infestation. Oral examination revealed two well‐circumscribed ulcers on either side of midline on the rostral hard palate.

Haematology documented low HCT 15.8 L/L (RI 25.0 to 45.0 L/L) and reticulocytosis 260,000/μL (RI 0/μL) consistent with a moderate, profoundly regenerative anaemia. Blood smear evaluation identified macrocytosis, anisocytosis and polychromasia. Serum biochemistry revealed increased urea concentration 13.6 mmol/L (RI 6.5 to 10.5 mmol/L) consistent with swallowed blood in the gastrointestinal tract, other changes were mild and not of concern.

A packed red blood cell transfusion was administered. PCV increased from 10% to 15% post transfusion. Under general anaesthesia, both major palatine arteries were ligated caudal to the ulcers using encircling 2‐0 polydioxanone sutures (PDS; Ethicon). The ulcers were cauterised with monopolar diathermy. The patient was discharged from the hospital 3 days post‐surgery with 0.5 mg/kg prednisolone to be given twice daily for pruritus for 1 week. Flea treatment was initiated prior to referral.

Minor oral haemorrhage was reported 7 months post‐surgery by the primary care practitioner via telephone call to the referral hospital. On clinical examination by the primary care practitioner, the cat was reported to be bright, alert and responsive, but no other information regarding vital parameters or clinical examination was available. The owners declined further assessment as this stage. Recommendations to the owners included keeping up with regular grooming for matted fur and ongoing preventative flea treatment to minimise overgrooming. No further follow‐up was available.

### Case 4

A 7‐year‐old, male neutered, DSH cat was presented with a 3‐day history of lethargy, anorexia and oral haemorrhage after returning home with blood around his mouth and neck. On presentation, the patient was mildly tachypnoeic (respiratory rate 44 breaths per minute), but other vital parameters were within normal limits. Physical examination identified pale mucous membranes, grade IV/VI heart murmur with an intermittent gallop sound and a flea infestation. Oral examination documented an approximate 8‐mm superficial ulcer on the right‐hand side of the rostral hard palate.

Haematology documented HCT 13.2 L/L (RI 25.0 to 45.0 L/L) and reticulocytosis 120,000 μL (RI 0/μL) consistent with a moderate, regenerative anaemia. Blood smear evaluation identified macrocytosis, anisocytosis and polychromasia. Serum biochemistry documented a mild increase in ALT activity 186 IU/L (RI 15 to 45 IU/L) but was otherwise considered unremarkable.

A packed red cell xenotransfusion was administered due to a seizure suspected to be a hypoxic event on the first day of hospitalisation. PCV post‐transfusion increased from 10% to 16%. The cat was taken to surgery, and the right major palatine artery was ligated using an encircling 3‐0 polydioxanone suture (PDS; Ethicon). The ulcer was cauterised with monopolar diathermy. The cat recovered without complications.

Additionally, during hospitalisation, the patient was identified as having congestive heart failure secondary to hypertrophic cardiomyopathy. Echocardiography identified marked bilateral cardiomegaly with mild concentric hypertrophy of the left ventricular posterior wall. Cardiac changes identified could be consistent with a compensatory mechanism activated in the face of the anaemia or cardiomyopathy. Repeat echocardiography in 3 months was recommended.

The patient was discharged 5 days post‐surgery with potassium supplementation (Kaminox; VetPlus, Chemeyes‐K; Chemeyes Pet Health), 2 mg of mirtazapine to be administered once daily if appetite is poor, 2 mg/kg frusemide (Frusol, Rosemont Pharmaceuticals Limited) three times daily ongoing for the treatment of congestive heart failure and flea treatment, imidacloprid (Advocate, Elanco).

Two months post‐surgery, the patient was reported to be doing well by the primary care practitioner with no recurrence of oral haemorrhage and resolution of the anaemia. Repeat haematology documented return of parameters to within normal limits and echocardiography by the primary care practitioner documented persistent marked left ventricular hypertrophy, so 1 mg/kg frusemide twice daily was continued ongoing and 18.75 mg clopidogrel once daily initiated. No further follow‐up is available.

## DISCUSSION

This article reviews four cases of Menrath ulcers in cats. All four cases had similar clinical signs consisting of oral haemorrhage, ulceration of the rostral hard palate, flea burden, pruritus and a moderate‐to‐severe regenerative anaemia. Flea infestation as a cause of pruritus has been reported as a presenting clinical sign in several case reports of Menrath ulcers (Bailey et al., 2007; Klinprathum et al., 2022; Mantelli et al., 2023). The similarities in the cases presented in this article and cases currently available in the literature are supportive of fleas, and associated pruritus, being a risk factor for the development of a Menrath ulcer in the cat. In addition to flea burden, regenerative anaemia was a common clinical finding, and as such, in a cat with a regenerative anaemia and flea infestation it would be pertinent to perform a full oral examination to check for the presence of a Menrath ulcer as a cause of haemorrhage, although the authors’ acknowledge that oral examination should be performed as part of a full clinical examination in any pruritic cat.

Biopsy findings of Menrath ulcers have been described as “hyperplastic, partially denuded epithelium with submucosal new vessel formation and moderate inflammatory infiltrate (Whitley et al., 2017)” and “hyperplastic stratified squamous epithelium with underlying dense connective tissue and interspersed granulation tissue, with no evidence of neoplasia (Bailey et al., 2007)”. In this case series, no biopsies were obtained during investigations or surgery. The appearance of Menrath ulcers has been described in the literature as well‐demarcated lesions located halfway between the dental arcade and midline on the rostral hard palate caudomedial to the canine tooth (Whitley et al., 2017), which is consistent with the location and appearance of Menrath ulcers identified and diagnosed in this case series.

Differential diagnoses for oral lesions in cats include eosinophilic granuloma complex (EGC), gingivostomatitis, oral neoplasia such as squamous cell carcinoma or fibrosarcoma and uraemic and viral ulceration (Clarke & Caiafa, 2014). Focal palatitis, formally known as focal palatine erosions (FPE) and first reported by Fitch and Fagan (1982), has been reported in captive cheetahs. The aetiology is now thought to be related to diet and foreign material trapped in palatal depressions. These ulcerations are typically more caudal than a Menrath ulcer. In these cases, oral haemorrhage has not been reported (Steenkamp et al., 2021). Eosinophilic granulomatous complex in cats can cause lesions of the hard palate and oral haemorrhage but typically have central white to yellow gritty deposits of degranulated eosinophils which differs from the usual presentation of a Menrath ulcer (Buckley & Nuttall, 2012). EGC usually has extra oral signs characterised as cutaneous reaction patterns and lesions can include indolent ulcers, eosinophilic plaques and eosinophilic granulomas. Diagnosis of EGC is normally based on clinical signs and cytology containing a large number of eosinophils (Buckley & Nuttall, 2012). In addition to EGC as a differential, oral neoplasia should be considered. The most common malignant oral neoplasia in cats is feline oral squamous cell carcinoma (FOSCC). These tumours are highly invasive and have metastatic potential. Common locations include sublingual/lingual region, mandible, maxilla, caudal pharynx, lip and buccal mucosa and tonsillar region (Bilgic et al., 2015). As these tumours can be found on the maxilla and have the potential to ulcerate, this differential should not be overlooked. Feline chronic gingivostomatitis (FCGS) causes severe oral pain and inflammatory lesions of the oral cavity. Diagnosis is made by histological confirmation (Soltero‐Rivera et al., 2024). Whilst the authors do not feel a biopsy is necessary for the diagnosis of a Menrath ulcer, it should be considered to exclude other differential diagnosis such as a neoplastic lesion or EGC lesion, which are treated very differently. Consideration should be given to the combination of presenting clinical signs and decision to biopsy should be made on a case‐by‐case basis. Risk of haemorrhage should be considered when performing a biopsy of a lesion on the hard palate. Uraemic ulcers would be expected to be accompanied by clinical signs and laboratory findings supportive of acute kidney injury (Monaghan et al., 2012). In the authors' opinion, a Menrath ulcer should be considered as a top differential in a case with an isolated ulcerated lesion of the rostral hard palate, unilateral or bilateral of either side of midline, with additional findings of oral haemorrhage, heavy flea burden, pruritus and regenerative anaemia.

The surgical management of the cases described in this case series differs slightly. As previously discussed, there is limited literature available on the best management of these ulcers; nevertheless, there are currently five surgical treatments described in the literature to treat Menrath ulcers including ligation of the major palatine artery ipsilateral to the ulcer (Hall, 2020; Menrath & Miller, 1995), cauterisation of the ulcer (Whitley et al., 2017; Wildgoose, 1990), suturing the edges of the ulcer using an appositional suture pattern (Fossum, 2019), reconstruction using a bipedicle mucoperiosteal flap (Bailey et al., 2007) and more recently described coblation of the ulcer (Mantelli et al., 2023).

The descriptions of ligation, cauterisation and suturing of the ulcer are minimal, and there are no references in the literature regarding complications associated with these techniques. The descriptions of the bipedicle mucoperiosteal flap and coblation techniques are more in depth and provide some insight into post‐operative complications and outcomes. The bipedicle mucoperiosteal flap technique describes blind ligation of the major palatine artery where it exits the palatine foramen of the hard palate and subsequent transposition of a bipedicle mucoperiosteal flap medially to cover the ulcer. Of the three surgical cases described in this case series, one case had further oral haemorrhage due to a new ulceration on the contralateral side to the previous surgical intervention (Bailey et al., 2007). Coblation‐ND is a low‐temperature thermal technique based on bipolar energy transformation with the use of saline. Advantages of this method include minimal thermal injury to local tissue. Coblation has been used to treat two ulcers in one patient without reported complications. No post‐operative haemorrhage was noted and improvement to haematological parameters was reported 2 days post‐surgery. Follow‐up 1 year later confirmed no further haemorrhage or complications (Mantelli et al., 2023).

The surgical techniques described in this series include unilateral blind ligation of the major palatine artery without cauterisation of the ulcer (case 1), blind ligation of unilateral and bilateral palatine arteries with cauterisation of the ulcer (cases 3 and 4) and cauterisation of the major palatine artery visualised via an open approach and subsequent suturing of the mucosal flap created (case 2). Within this case series, one cat (case 1) had a severe post‐operative complication requiring revision surgery and one cat (case 3) had a mild post‐operative complication. Case 1 had persistent severe oral haemorrhage after blind ligation of the left major palatine artery requiring blood transfusion and revision surgery. In case 3, the ulcer was cauterised in addition to blind ligation of the major palatine artery but also had further oral haemorrhage suggesting that cauterisation in addition to ligation of the artery is not completely effective at preventing repeat haemorrhage; however, the bleeding in this case was minor and did not require any revision surgery. The same technique was performed in case 4 without reported complication. Case 2 developed a palatal defect post‐operatively, which has not previously been reported in the literature. This complication has not been classified as there was limited follow‐up available. It is possible that this patient went on to suffer complications of a palatal defect such as chronic rhinitis or an aspiration event in which case surgical intervention may have been indicated, and therefore, this would be classified as a severe complication. The client was noted to have financial constraints, which may have influenced the decision to monitor and therefore the overall classification of this post‐operative complication.

Case 1 was initially managed conservatively, but further clinically significant oral haemorrhage ensued rapidly. Conservative management is a risk and currently not recommended by the authors. The client should be made aware that marked, potentially life‐threatening haemorrhage could occur if left untreated. Therefore, it is the authors’ recommendation that Menrath ulcers are surgically managed. The patient must first be evaluated for anaemia and cardiovascular stability. Based on the literature and this case series ligation of the major palatine artery, either unilaterally if the ulcer is one sided or bilaterally if both sides of the hard palate are ulcerated, is indicated. Ligation can be performed blindly with knowledge of the anatomy of the hard palate and the course of the major palatine arteries. Care should be taken when using electrocautery close to palatal bone as thermal necrosis of the bone shelf may result in a palatal defect as seen in case 2. A monofilament synthetic absorbable suture material is recommended, such as polydioxanone, for ligatures. For suturing of the oral cavity, monofilament absorbable suture such as Poliglecaprone 25 (Monocryl, Ethicon) is recommended due to reduced drag on tissues and its relatively quick complete absorption time compared with other monofilament absorbable sutures available. The newly described Coblation‐ND technique (Mantelli et al., 2023) offers a less traumatic approach for local tissues, but electrocautery is likely more readily available.

In combination with medical management of the patient in hospital and surgical management of the Menrath ulcer, it is imperative to manage pruritus as the cause for the erosion. Patients should be treated for fleas with effective anti‐parasiticides lifelong. Treatment should extend to all pets in the household and effective treatment of the environment to prevent reinfestation. In the interim to control the pruritus associated with flea allergy, short courses of steroids and antihistamines are the mainstay of treatment. The patients treated with the bipedicle mucoperiosteal flap (Bailey et al., 2007) also had Elizabethan collars for several days post‐operatively to further prevent repeated trauma to the healing ulcer, this may provide additional reassurance whilst medication takes effect.

All cases in this article survived to discharge and were reported to be doing well 1 to 8.5 months post‐surgery. Each case had variable follow‐up, and with the retrospective nature of this case series, it was not possible to gather long‐term outcomes for these patients. The outcomes reported are similar to the outcomes of other cases available in the literature. Despite the limitations, it could be concluded that the prognosis for Menrath ulcer is good provided further arterial haemorrhage is prevented irrespective of the surgical method used. It is unknown how often cats present with fatal arterial bleeding prior to diagnosis, since fatal oral bleeding could be wrongfully attributed to other causes, for example road‐traffic accidents, head trauma, haemoptysis secondary to immune‐mediated thrombocytopenia, disseminated intravascular coagulation and severe destructive rhinitis.

The case series has several limitations. First, being a case series consisting of only four cats, it is challenging to provide representative statistics regarding complications and prognosis of cats with Menrath ulcers. Second, the retrospective nature of the study led to records being incomplete and specifics regarding surgical technique were not always recorded. As mentioned previously, there is no information regarding the prevalence of Menrath ulcers in the cat population, and it is possible that cases are rare or missed either due to minor bleeding and swallowing of the blood, or due to catastrophic arterial bleeding leading to death.

At the time of writing, this case series is the first to describe an acquired palatal defect and recurrence of oral haemorrhage from the original Menrath ulcer as complications of surgical management. This is also the largest case series to date, and the literature regarding Menrath ulcers is limited. A Menrath ulcer is an important differential in cats presenting with moderate‐to‐severe regenerative anaemia, oral ulceration, oral haemorrhage and pruritus. The mainstay of surgical management involves ligation of the major palatine artery on the affected side. Surgical intervention does not completely prevent the risk of further arterial haemorrhage and effective control of pruritus usually caused by fleas is imperative to reduce risk of further ulceration and haemorrhage. Despite complications, the prognosis after surgical intervention in the cases described in this article and other cases reported in the literature is good. Veterinarians and clients should be aware of the possibility of recurrence of ulceration and oral haemorrhage and risk of development of a palatal defect post‐intervention.

### Author contributions

**K. Whybrow:** Data curation (equal); formal analysis (equal); investigation (equal); methodology (equal); project administration (lead); writing – original draft (lead); writing – review and editing (equal). **T. Hernon:** Conceptualization (equal); project administration (equal); supervision (equal); writing – review and editing (equal). **M. Pilot:** Conceptualization (lead); data curation (equal); project administration (equal); supervision (equal); writing – review and editing (equal).

### Conflict of interest

None of the authors of this article has a financial or personal relationship with other people or organisations that could inappropriately influence or bias the content of the paper.

## Data Availability

The data that supports the findings of this study are available from the corresponding authors, upon reasonable request.
